# Supplementary material for: Lattice-mismatch-free construction of III-V/chalcogenide core-shell heterostructure nanowires
Source: Nat Commun. 2023 Nov 18;14:7480. doi: 10.1038/s41467-023-43323-x (PMC10657406; doi:10.1038/s41467-023-43323-x)
Supplement: Supplementary file 1 — Supplementary Information [file 41467_2023_43323_MOESM1_ESM.pdf]

Supplementary Information For

Lattice-mismatch-free construction of III-V/chalcogenide core-shell heterostructure nanowires

Fengjing Liu[1], Xinming Zhuang[1], Mingxu Wang[1], Dongqing Qi[2], Shengpan Dong[3], SenPo Yip[4], Yanxue Yin[1], Jie Zhang[1], Zixu Sa[1], Kepeng Song[2,], Longbing He[3], Yang Tan[1], You Meng[5], Johnny C. Ho[4,5,*], Lei Liao[6], Feng Chen[1], and Zai-xing Yang[1,*]*

[1] School of Physics, State Key Laboratory of Crystal Materials, Shandong University, Jinan, 250100, China

[2] School of Chemistry and Chemical Engineering, Shandong University, Jinan, 250100, China

[3] SEU-FEI Nano-Pico Center, Key Lab of MEMS of Ministry of Education, Collaborative Innovation Center for Micro/Nano Fabrication, Device and System, Southeast University, Nanjing, 210096, China

[4] Institute for Materials Chemistry and Engineering, Kyushu University, Fukuoka, 816-8580, Japan

[5] Department of Materials Science and Engineering, City University of Hong Kong, Hong Kong, 999077, China

[6] Key Laboratory for Micro-Nano Optoelectronic Devices of Ministry of Education, School of Physics and Electronics, Hunan University, Changsha, 410082, China

*Address Correspondence to K.P. Song (kpsong@sdu.edu.cn), J.C. Ho (johnnyho@cityu.edu.hk), and Z.-x. Yang (zaixyang@sdu.edu.cn).

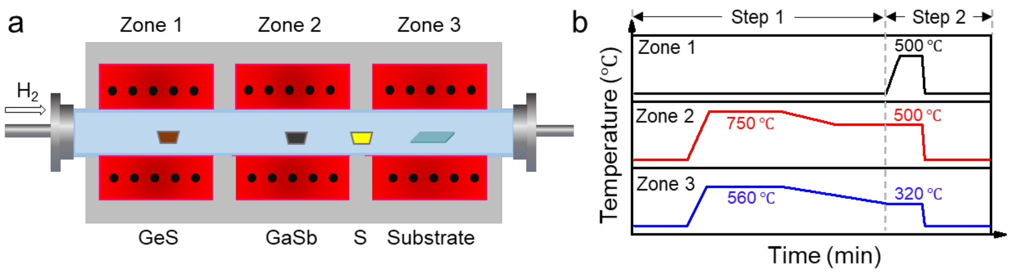

Supplementary Fig. S1 Construction of GaSb/GeS core-shell heterostructure NWs. a, Schematic of the three-temperature-zone tube furnace. **b**, Illustration of the heating sequence and heating time of the three zones for the growth of GaSb/GeS core-shell heterostructure NWs.

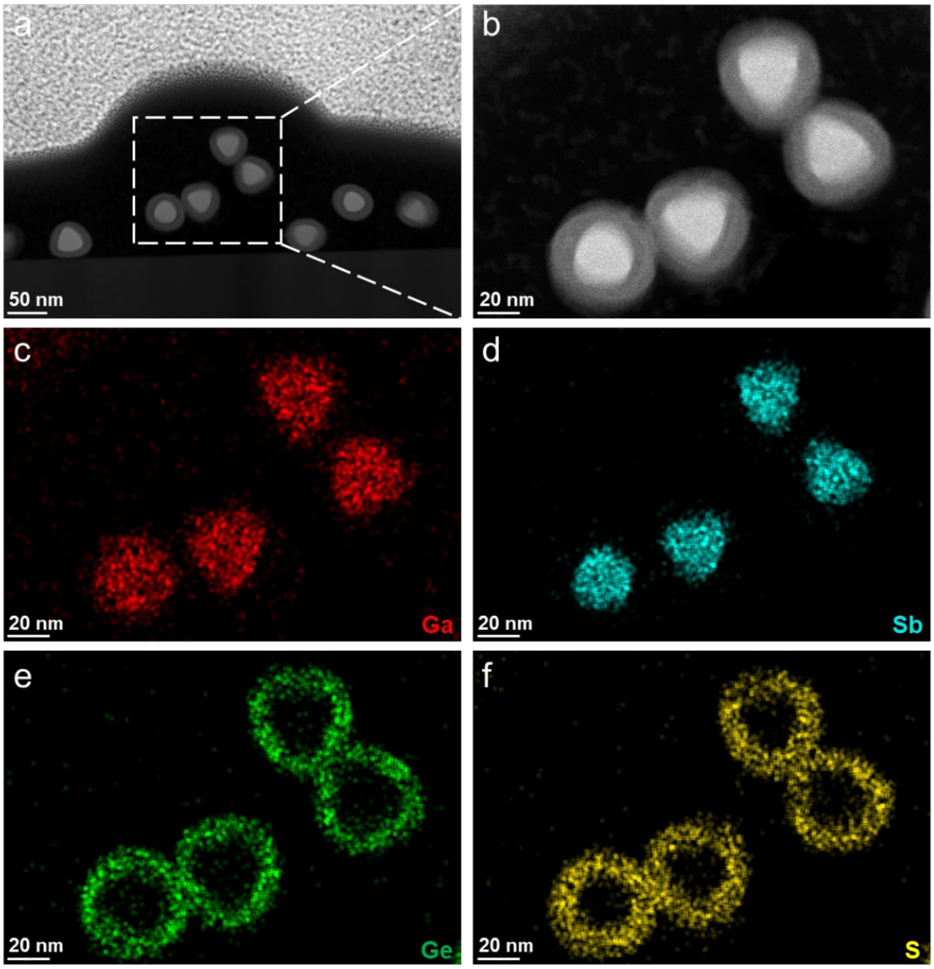

Supplementary Fig. S2 Homogeneity of the as-constructed GaSb/GeS core-shell heterostructure NWs. a,b, Low-magnification cross-sectional HAADF STEM images of the as-constructed GaSb/GeS core-shell heterostructure NWs. **c-f**, EDS mapping images of Ga, Sb, Ge, and S.

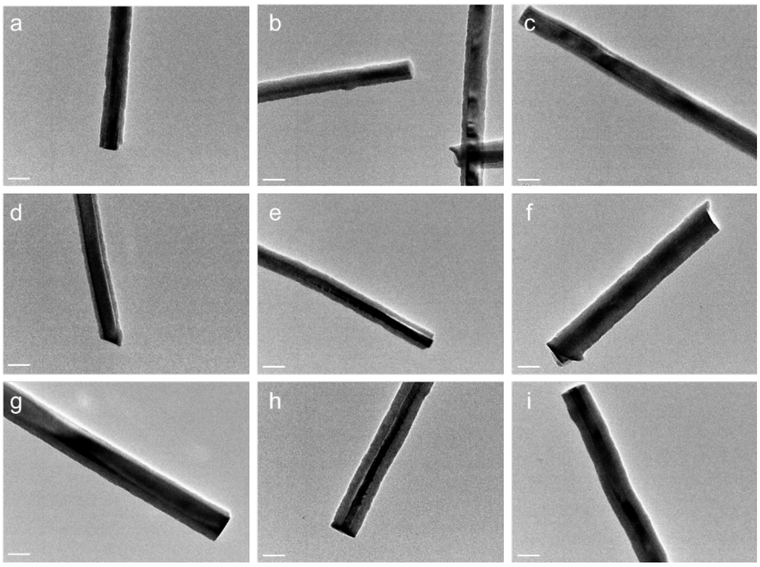

Supplementary Fig. S3 TEM images of as-constructed GaSb/GeS core-shell heterostructure NWs. **a-i**, TEM images of as-constructed GaSb/GeS core-shell heterostructure NWs at the end sections. All the scale bars are 50 nm.

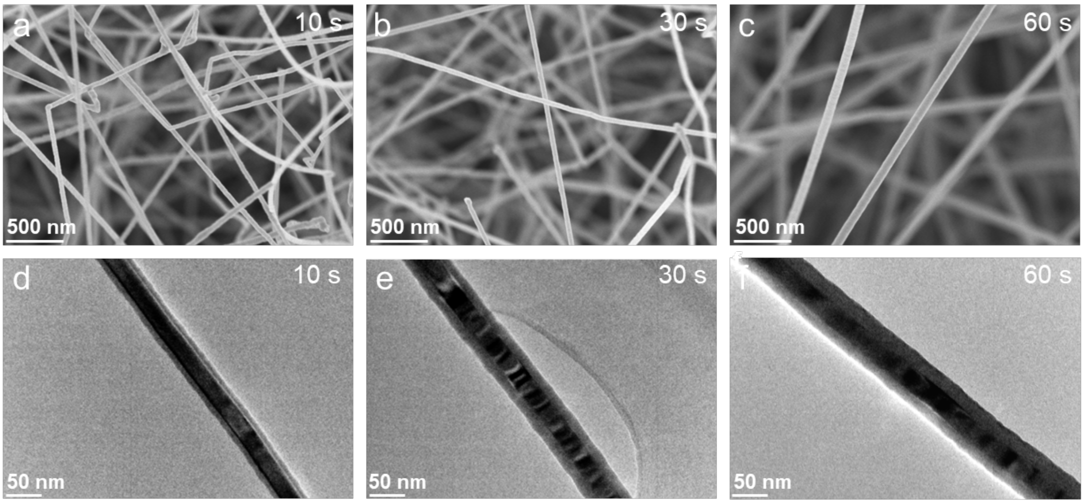

Supplementary Fig. S4 Controllable construction of GaSb/GeS core-shell heterostructure NWs. SEM images (**a-c**) and TEM images (**d-f**) of the as-constructed GaSb/GeS core-shell heterostructure NWs with different growth times of GeS shell.

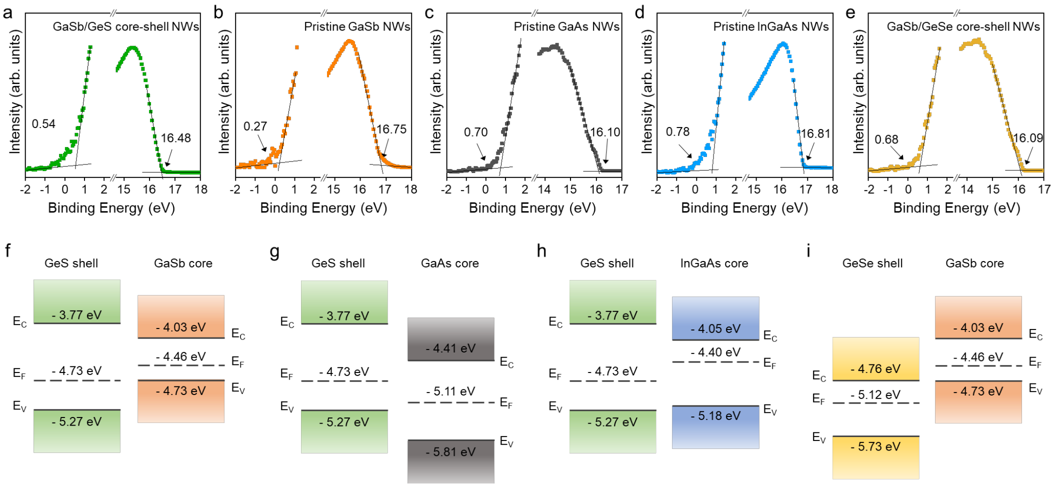

Supplementary Fig. S5 Band alignments of as-constructed core-shell heterostructure NWs. a-e, UPS of GaSb/GeS core-shell heterostructure NWs, pristine GaSb NWs, pristine GaAs NWs, pristine InGaAs NWs, and GaSb/GeSe core-shell heterostructure NWs, respectively. The solid black lines mark the baselines and the tangents of the curves. The intersections of the tangents with the baselines indicate the edges of UPS. **f-i**, Type-I band alignment of GaSb/GeS heterostructure, Type-II band alignments of GaAs/GeS heterostructure, InGaAs/GeS heterostructure, and GaSb/GeSe heterostructure.

Ultraviolet photoelectron spectroscopy (UPS, ESCALAB XI+, ThermoFisher) is adopted to evaluate the band structures of as-constructed core-shell heterostructure NWs. The band structures are deduced from UPS of pristine GaSb NWs, GaSb/GeS core-shell heterostructure NWs, pristine GaAs NWs, pristine InGaAs NWs, and GaSb/GeSe core-shell heterostructure NWs. According to the linear intersection method, the valence band (E_V) values of GaSb, GeS, GaAs, InGaAs, and GeSe are calculated as - 4.73 eV, - 5.27 eV, - 5.81 eV, - 5.18 eV, and - 5.73 eV, respectively, by subtracting the width of He I UPS from the excitation energy of 21.21 eV. Meanwhile, the work functions (E_F) of GaSb, GeS, GaAs, InGaAs, and GeSe are calculated as - 4.46 eV, - 4.73 eV, - 5.11 eV, - 4.40 eV, and - 5.12 eV, respectively, by adding E_v to the second electron cutoff energy. According to the bandgap values of GaSb (0.70 eV), GeS (1.50 eV), GaAs (1.40 eV), InGaAs (1.13 eV), and GeSe (0.97 eV) reported in the literatures[1-4], the conduction band (E_c) values of GaSb, GeS, GaAs, InGaAs, and GeSe are calculated as - 4.03 eV, - 3.77 eV, - 4.41 eV, - 4.05 eV, and - 4.76 eV, respectively. In this case, the band alignment diagrams of as-constructed GaSb/GeS, GaAs/GeS, InGaAs/GeS, and GaSb/GeSe core-shell heterostructure NWs are drawn approximated. Obviously, the heterostructures with expected heterostructure type, such as type-I of GaSb/GeS and type-II of GaAs/GeS, InGaAs/GeS and GaSb/GeSe are constructed successfully.

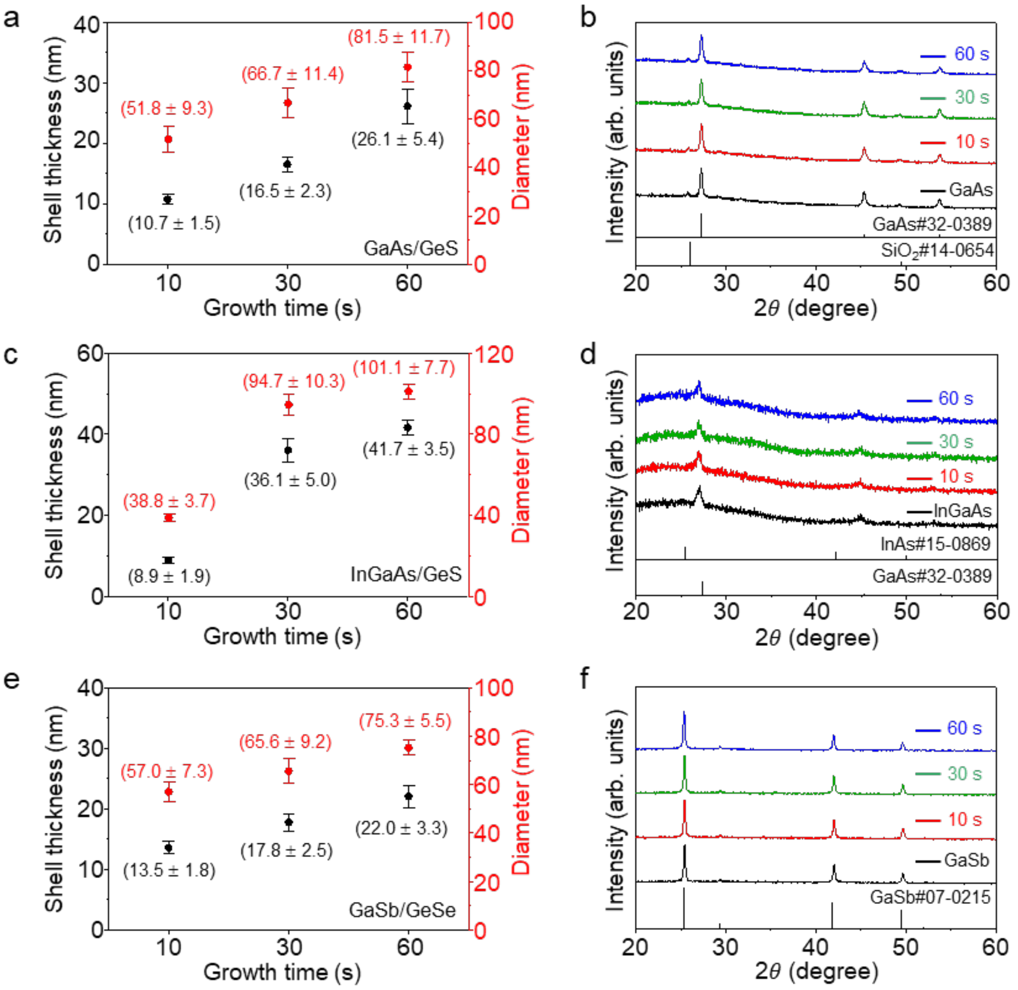

Supplementary Fig. S6 Construction of the GaAs/GeS, InGaAs/GeS, and GaSb/GeSe core-shell heterostructure NWs. **a**,**b**, Diameter, shell thickness statistics and XRD patterns of GaAs/GeS core-shell heterostructure NWs, respectively. **c**,**d**, Diameter, shell thickness statistics and XRD patterns of InGaAs/GeS core-shell heterostructure NWs, respectively. **e**,**f**, Diameter, shell thickness statistics, and XRD patterns of GaSb/GeSe core-shell heterostructure NWs, respectively. The diameter and shell thickness statistics are reproduced on 25 samples and the error bar represents the standard deviation.

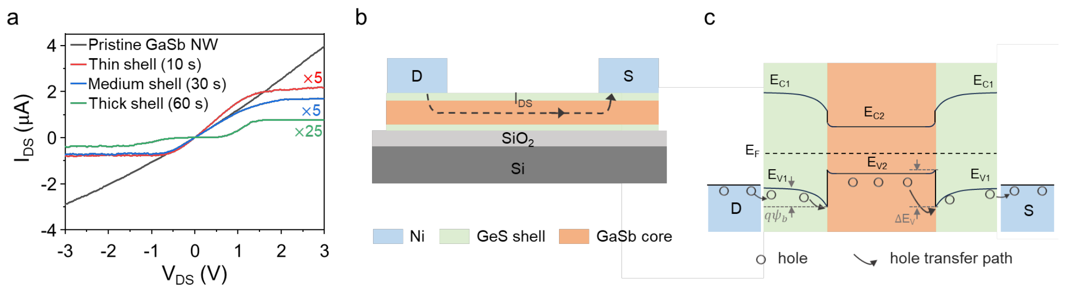

Supplementary Fig. S7 Electrical properties of GaSb/GeS core-shell heterostructure NWs. a, I-V curves of the pristine GaSb NW and GaSb/GeS core-shell heterostructure NWs with different shell thicknesses. **b**, Schematic of NW MSM photodetector. **c**, Holes transport in the GaSb/GeS core-shell heterostructure NW.

Fig. S7 shows the electrical properties of as-constructed GaSb/GeS core-shell heterostructure NWs. From the I-V curves of Fig. S7a, the I_{DS} of pristine GaSb NW MSM photodetector changes linearly with V_{DS}, demonstrating the typical Ohmic contacts between Ni electrodes and GaSb NW. With a V_{DS} of 3V, the I_{DS} is 3.96 μA. For the GaSb/GeS core-shell heterostructure NWs MSM photodetectors, the I_{DS} are significantly reduced compared to that of pristine GaSb NW MSM photodetector. At the same time, the saturated I_{DS} are observed under large bias voltages. The saturated I_{DS} decrease from 304 to 217 and 12 nA for thin, medium, and thick shell NWs. In short, with the increase of shell thickness, the I_{DS} of as-studied MSM photodetectors decrease, indicating the GaSb cores act as the main conductive channels (as shown in Fig. S7b), and the GeS shells limit the currents.

To further illustrate the current limiting mechanism, a band structure model is depicted in Fig. S7c for demonstrating the holes transport process at the interface of GaSb and GeS. Under a forward bias voltage, the holes inject from drain electrode to the main conductive channel of GaSb core and are collected at source electrode, as shown in Fig. S7b. The holes pass across the GeS shell during both the injection and collection processes. As shown in Fig. S7c, a forward bias heterojunction and a reverse bias heterojunction are formed at the injection and collection regions, which block the holes transport and result in the decreased currents compared to pristine GaSb NW MSM photodetector. At thermal equilibrium, the barrier of the forward bias heterojunction (due to the Fermi level coincident) is $q\psi_b$ (q is the electronic charge and ψ_b is electrostatic potential), and the barrier of the reverse bias heterojunction (due to the valence band offset) is ΔE_V (E_V is the valence band edge)[5,6]. Obviously, with the increase of bias voltage, the barrier of the forward heterojunction of $q\psi_b$ at injection region will decrease, which will benefit to the holes transport, resulting in the increase of I_{DS}. At the same time, the barrier of the reverse heterojunction at collection region keeps ΔE_V. In this case, the saturated I_{DS} is observed at a large bias voltage. It is worth mentioning that in addition to the

classical thermionic emission, tunneling or defect-assisted tunneling also plays role on the holes injection and collection processes[7,8]. With a thin shell, the barrier is possible thin and low, benefiting to the holes tunneling process. As a result, with the increase of shell thickness, the I_{DS} decreases.

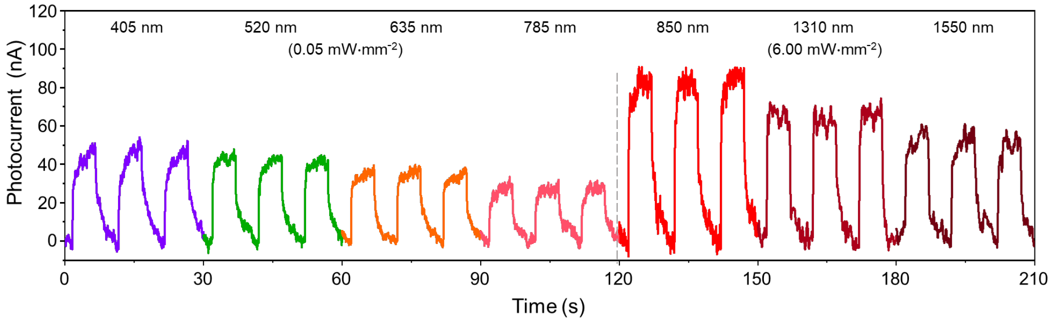

Supplementary Fig. S8 Broad-spectrum photodetection performance of pristine GaSb NWs.

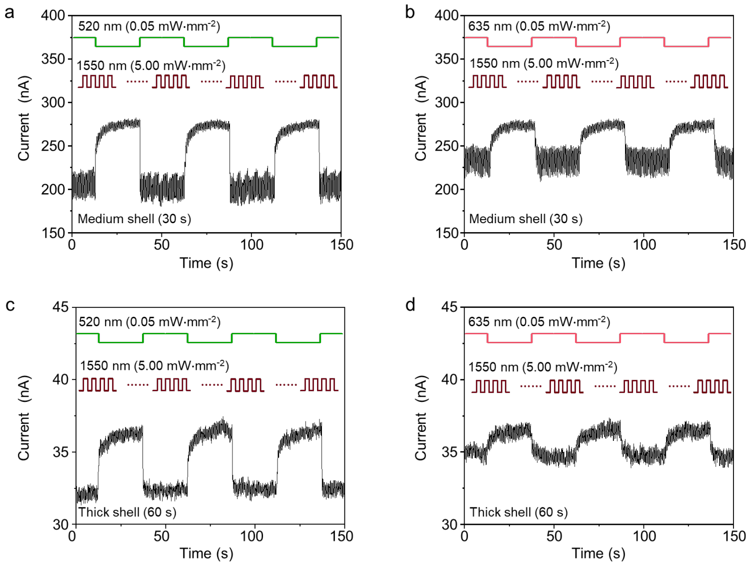

Supplementary Fig. S9 Visible light-assisted infrared photodetection performance of GaSb/GeS core-shell heterostructure NWs. a,b, The visible light-assisted infrared photodetection performance of NWs with a medium shell, where 520 nm and 635 nm light act as assisted light, respectively. **c,d**, The visible light-assisted infrared photodetection performance of NWs with a thick shell, where 520 nm and 635 nm light act as assisted light, respectively.

Based on the mechanism of visible light-assisted behavior, both 520 nm and 635 nm lights also can improve the infrared photodetection performance of photodetector fabricated by GaSb/GeS core-shell heterostructure NWs with an appropriate shell thickness. As shown in Fig. S9a, with a medium shell, when assisted light of 520 nm is on, the dark current of the infrared photodetector decreases from 269 nA to 187 nA. At the same time, the photocurrent increases from 10 nA to 27 nA. When auxiliary light of 635 nm is on (Fig. S9b), the dark current of the infrared photodetector decreases from 266 nA to 216 nA, and the photocurrent increases from 11 nA to 22 nA. For photodetector with a thick shell, with the assistance of visible lights of 520

nm and 635 nm, the dark currents of the photodetectors for infrared light are reduced, as shown in Fig. S9c and S9d. But barely any improvement of infrared photodetection currents is observed, similar to 405 nm assisted light (Fig. S15b).

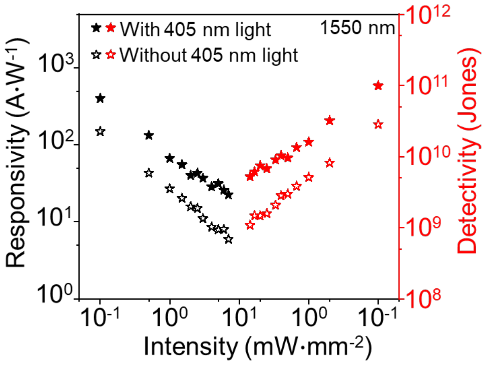

Supplementary Fig. S10 Responsivity and detectivity of as-fabricated GaSb/GeS core-shell heterostructure NWs under the illumination of 1550 nm laser with and without 405 nm light.

The R can be defined as $R = I_{ph}/(P \cdot A)$, in which I_{ph} is the photocurrent, P is the incident power density, and A is the effective irradiated area of the corresponding photodetector. D^* can be defined as $D^* = R \cdot A^{1/2}/(2e \cdot I_{dark})^{1/2}$, where e is the electronic charge, and I_{dark} is the dark current.

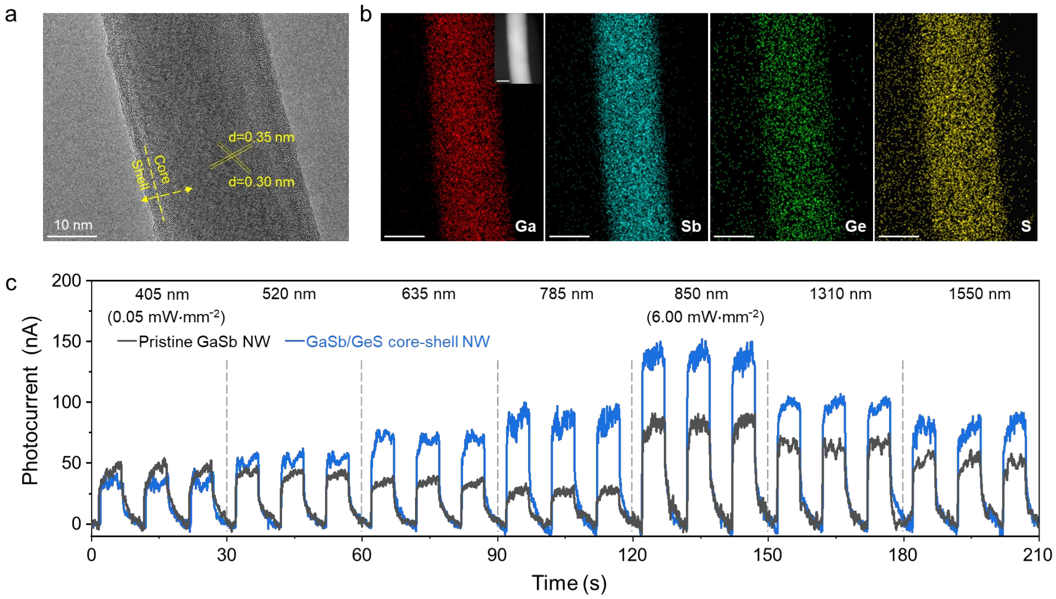

Supplementary Fig. S11 Construction of GaSb/GeS core-shell heterostructure NWs with ultra-thin GeS shells and their photodetection behaviors. a, HRTEM image of GaSb/GeS core-shell heterostructure NW with ultra-thin shell. **b**, EDS elemental mapping images of Ga, Sb, Al, O. The inset is the corresponding scanning transmission electron microscopy (STEM) image. All the scale bars are 20 nm. **c**, Broad-spectrum photodetection behaviors of pristine GaSb NWs and GaSb/GeS core-shell heterostructure NWs with ultra-thin shell. The laser intensities from 405 nm to 785 nm and 850 nm to 1550 nm are 0.05 mW·mm^{-2} and 6.00 mW·mm^{-2}, respectively.

The ultra-thin GeS shell also grows on the surfaces of GaSb NWs for studying the passivation effect. From the HRTEM image of Fig. S11a, with a growth time of 1 s, the shell of as-constructed GaSb/GeS core-shell heterostructure NWs is around 2 nm. As shown in the EDS mapping images of Fig. S11b, Ga and Sb dominate the NW core. At the same time, Ge and S dominate the shell. This finding is in line with the result of GaSb/GeS core-shell heterostructure NW with a thicker shell, as shown in Fig. 1. From the broad-spectrum photodetection behavior of Fig. S11c, it is found that GaSb/GeS core-shell heterostructure NW exhibits larger photocurrent compared to that of pristine GaSb NW, which is attributed to the surface passivation effect of ultra-thin GeS shell. At the same time, due to the negative photoresponse caused by the GeS shell, a reduced photocurrent is also observed at the near ultraviolet waveband of 405 nm. In short, the amorphous chalcogenide shells not only overcome the lattice mismatch, but also passivate the surface charge trappings of III-V NWs.

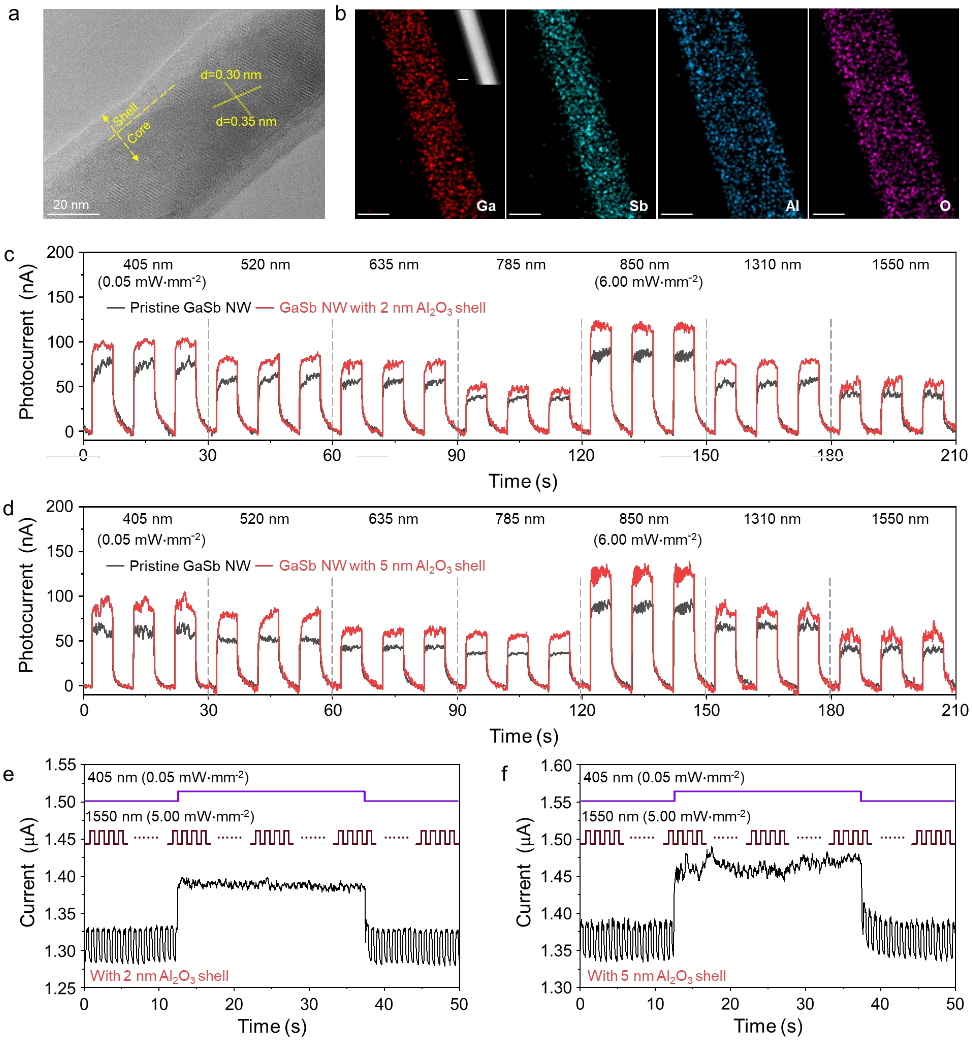

Supplementary Fig. S12 Construction of GaSb/Al_2O_3 core-shell NWs and their photodetection behaviors. a, HRTEM image of GaSb/Al_2O_3 core-shell NW. **b**, EDS elemental mapping images of Ga,

Sb, Al, O. All the scale bars are 20 nm. **c, d**, Broad-spectrum photodetection behaviors of pristine GaSb NWs and GaSb/Al_2O_3 core-shell NWs with 2 nm and 5 nm Al_2O_3 shell, respectively. The laser intensities from 405 nm to 785 nm and 850 nm to 1550 nm are 0.05 mW·mm^{-2} and 6.00 mW·mm^{-2}, respectively. **e**, **f**, Infrared photodetection behaviors of GaSb/Al_2O_3 core-shell NWs with 2 nm and 5 nm Al_2O_3 shells under the illuminations of visible light, respectively. The laser intensities of 405 nm and 1550 nm are 0.05 mW·mm^{-2} and 5.00 mW·mm^{-2}, respectively.

Beyond amorphous GeS, the larger bandgap Al_2O_3 is also attempted to passivate the surface charge trappings of GaSb NWs, as shown in Fig. S12. The Al_2O_3 shells grow on the surfaces of GaSb NWs by atomic layer deposition method. The HRTEM image and EDS mapping images of Fig. S12a-b show that GaSb/Al_2O_3 core-shell heterostructure NW with a shell of 5 nm is successfully constructed. Ga and Sb dominate the NW core. On the other hand, Al and O dominate the shell. From the broad-spectrum photodetection behaviors of Fig. S12c-d, it is found that GaSb NWs with the Al_2O_3 shells of 2 nm and 5 nm both exhibit larger photocurrents compared to the pristine GaSb NW, which is attributed to the surface passivation effect of Al_2O_3 shells. Furthermore, the visible light-assisted infrared photodetection behaviors are also studied in Fig. S12e-f. When the visible light is on, the infrared photodetection currents can be distinguished hardly. This result can be attributed to the fact that a large number of carriers generated by visible light act as background carriers for infrared photodetection, which leads to the serious recombination of photogenerated carriers (generated by 1500 nm laser)[9,10]. The results show that the epitaxial larger bandgap Al_2O_3 shells can passivate the surface charge trappings of GaSb NWs effectively. Compared to the as-constructed III-V/chalcogenide core-shell heterostructure NWs, wavelength-dependent bi-directional photodetection behavior, visible light-assisted infrared photodetection behavior, and faster response times are not observed in GaSb/Al_2O_3 core-shell heterostructure NWs.

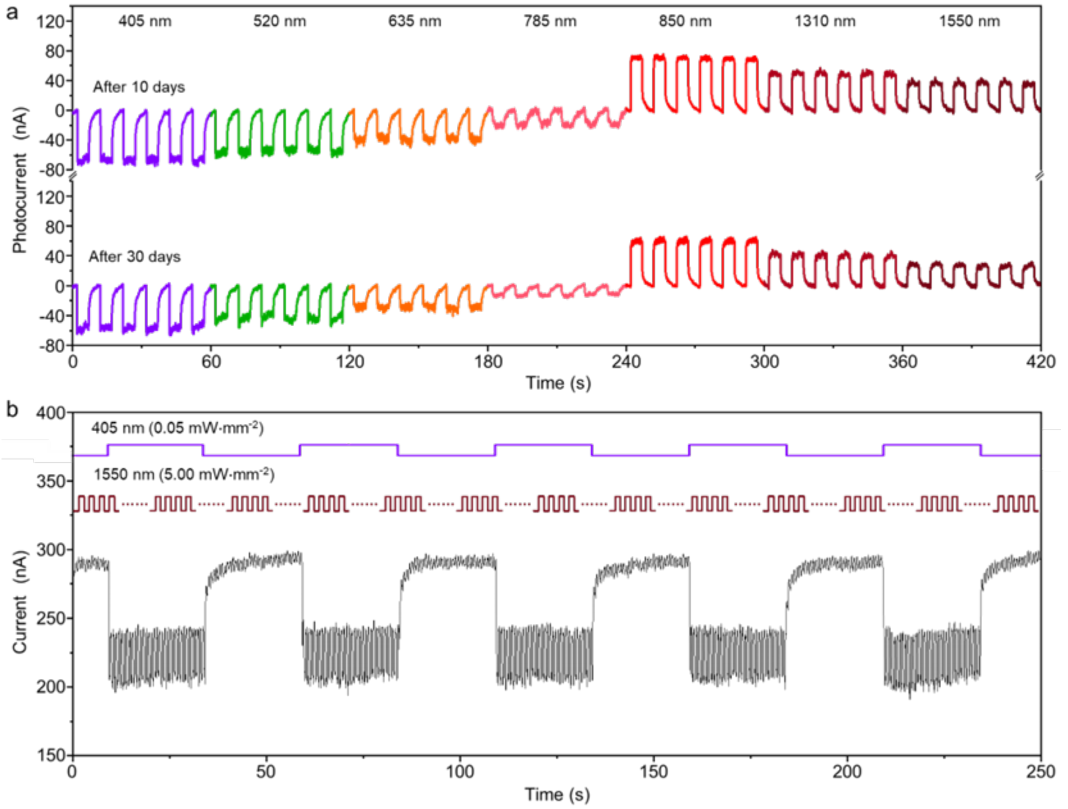

Supplementary Fig. S13 Stability of as-fabricated GaSb/GeS core-shell heterostructure NW photodetector. a, Broad-spectrum photodetection behavior of GaSb/GeS core-shell heterostructure NW after being stored in an atmospheric environment for 10 days and 30 days. The laser intensities from 405 nm to 785 nm and from 850 nm to 1550 nm are 0.05 mW·mm^{-2} and 6.00 mW·mm^{-2}, respectively. **b**, Visible light-assisted infrared photodetection performance of GaSb/GeS core-shell heterostructure NW after being stored in an atmospheric environment for 30 days.

As presented in Fig. S13a, the as-fabricated photodetector still exhibits stable wavelength-dependent bi-directional photodetection behavior, which displays a negative photoresponse in the wavelength of 405-785 nm and a positive photoresponse in the wavelength of 850-1550 nm after being stored in an atmospheric environment for 10 days and 30 days. The photocurrent attenuations are less than 10% and 20% for 10 days and 30 days, respectively. As shown in Fig. S13b, the as-fabricated photodetector also shows the visible light-assisted infrared photodetection behavior after being stored in an atmospheric environment for 30 days. It is found that when the assisted light of 405 nm is on, the dark current of the infrared photodetector is significantly suppressed, and the photocurrent increases obviously. In short, benefitting the as-constructed core-shell nanostructure, the as-fabricated photodetector exhibits stable, repeatable, and robust photodetection performance, which promises the application in further optoelectronic devices.

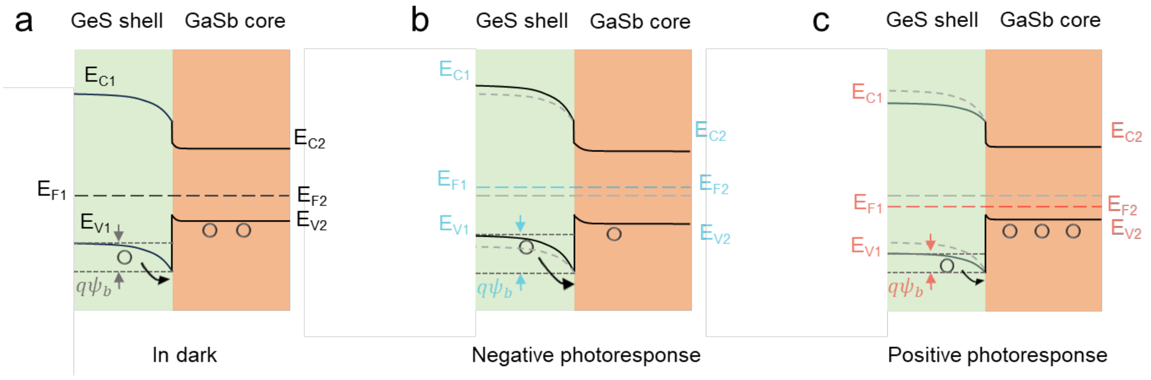

Supplementary Fig. S14 The photo-generated holes transport of GaSb/GeS core-shell heterostructure NW. a, Schematic of holes transport in dark. Schematics of holes transport with the decreased (b) and increased (c) hole concentration in GaSb core, respectively.

Because both the carrier injection and collection pass across the GeS shell, it also plays important role on the transport of photo-generated carriers at the contacts. As discussed in the manuscript, the electron-hole pairs generated in the channel of core-shell heterostructure NWs will be collected at contacts under a bias voltage. The photo-generated holes also pass across the GeS shell both at drain and source contacts. Obviously, the forward barrier of $q\psi_b$ dominates the transport of photo-generated holes (the reverse bias heterojunction is always ΔE_V). As shown in Fig. S14, the hole concentration of GaSb core seriously affect the hole transport at the contact[11]. When the hole concentration of GaSb core decreases, the Fermi level of E_{F2} will rise (far away from the valence band), resulting in an increased hole transport barrier of $q\psi_b$ (Fig. S14b). The increased barrier will block the transport of holes, leading to a decreased current (negative photoresponse). On the contrary, when the hole concentration of GaSb core increases, the E_{F2} will fall (close to the valence band), resulting in a decreased hole transport barrier (Fig. S14c). The decreased barrier will promote the transport of holes, leading to an increased current (positive photoresonse). Additionally, the thickness of GeS shell also has an important impact on the hole transport in photorespose behavior, owing to the tunneling. From the dynamic photoresponse results in Fig. 4 and Fig. S15, with the shell thickness increases from 11.3 ± 2.0 nm to 14.7 ± 1.9 nm and 19.5 ± 4.7 nm, the dark current decreases from 301 nA to 256 nA and 28 nA, and the photocurrent decreases from 73 nA to 64 nA and 6 nA under the illumination of 850 nm light.

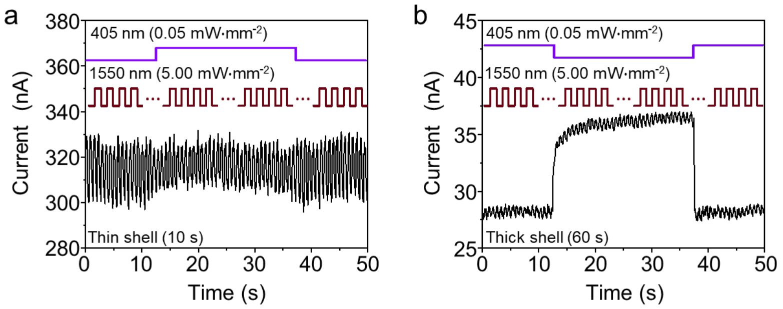

Supplementary Fig. S15 The visible light-assisted infrared photodetection performance of NWs with a thin shell (a) and a thick shell (b).

The shell thickness also plays an important role in visible light-assisted behavior. As shown in Fig. S15a, when the visible light (405 nm) is on, the infrared photodetection currents are suppressed in the thin shell core-shell NWs photodetector. This suppression is because the absorption of visible light is dominated by the GaSb core in the thin shell core-shell NWs. In this case, the photogenerated carriers generated by 1500 nm laser are annihilated in the large number of carriers generated by visible light, resulting in the suppressed photocurrents. With a medium shell, the visible light is mainly absorbed by the GeS shell. When the visible light is on, the dark current of the photodetector for infrared light is reduced due to the photogenerated electrons in the GeS shell recombining with the free holes in the GaSb core, as shown in Fig. 4e. Meanwhile, the trapped photogenerated holes in the GeS shell lead to the field-effect passivation[12,13], which would inhibit the recombination of the infrared light excited electron-hole pairs in the GaSb core, ultimately leading to the improvement of infrared photodetection currents. With a thick shell, the dark current is also decreased with visible light, as shown in Fig. S15b. But barely any improvement of infrared photodetection current is observed, possibly due to the thick shell weakens the field-effect passivation effect.

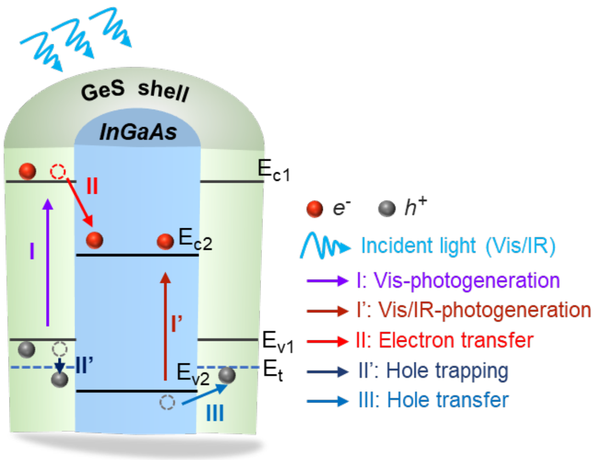

Supplementary Fig. S16 Schematic for type-II InGaAs/GeS core-shell heterostructure NWs photodetector.

As shown in Fig. S16, with the bandgap of 1.13 eV for the InGaAs core, both the InGaAs core and GeS shell can absorb the visible light (< 785 nm) and generate electron-hole pairs (processes I and I'). Under the illumination of visible light, the photogenerated electrons in GeS would transfer to the InGaAs core, driven by the built-in electric field of the band offset of the conduction band (E_C) (process II). In contrast, the photogenerated holes are trapped in the shell (process II'). The injected electrons would then increase the majority carrier (electron) concentration of the n-type InGaAs core. In the case of the InGaAs core, the photogenerated holes transfer to the GeS shell, which is induced by the built-in electric field of the band offset of the valence band (E_V) (process III). The effective separation of photogenerated electron-hole pairs leads to the significant photocurrent improvement of the InGaAs/GeS core-shell heterostructure NW in the visible waveband (Fig. 5a). Under the illumination of near-infrared light, only the InGaAs core absorbs the light, generating the electron-hole pairs (process I'). Owing to the efficient spatial separation of photogenerated carriers driven by the built-in electric field, the lifetime of photogenerated carriers in the InGaAs/GeS core-shell heterostructure NW is much longer than that in the pristine InGaAs NW, resulting in the improvement of photodetection performance in the near-infrared waveband (Figs. 5a&b). In addition, the built-in electric field of the heterojunction also drives the charges to passivate the defects at the interface, improving the response speed (Fig. 5c)[14].

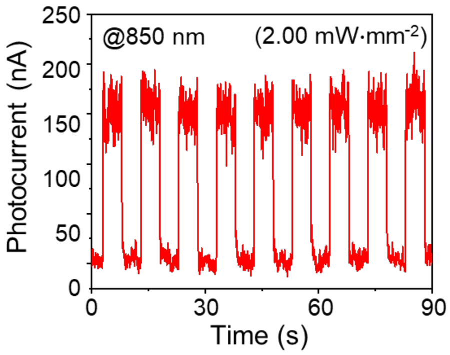

Supplementary Fig. S17 The temporal photoresponse characteristics of the array imaging unit under the illumination of 850 nm laser.

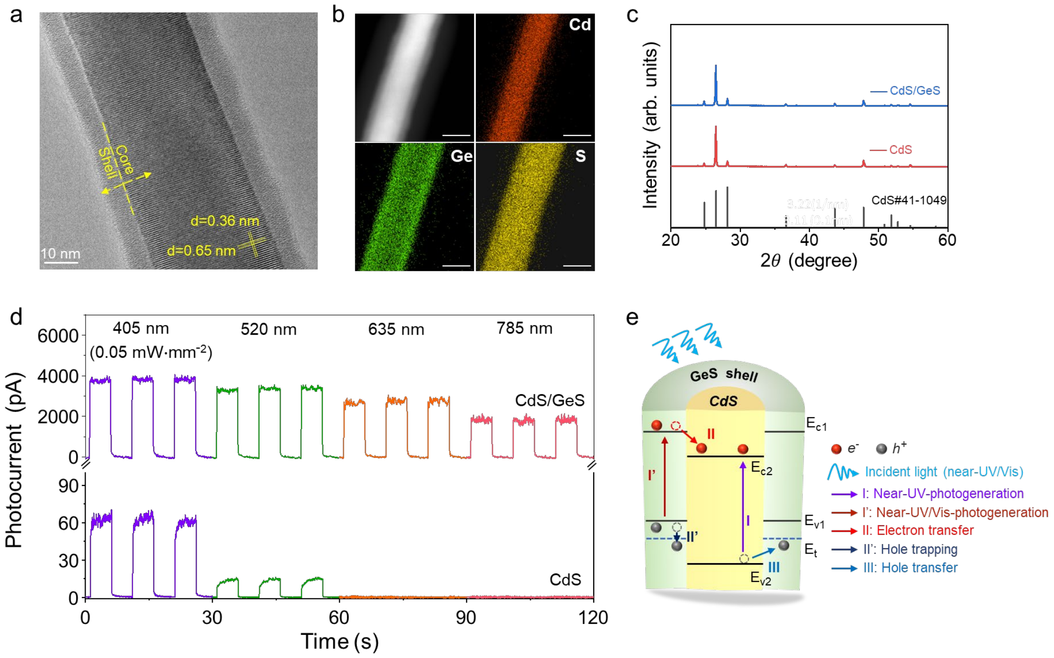

Supplementary Fig. S18 Lattice-mismatch-free construction of CdS/GeS core-shell heterostructure NWs and their photodetection behaviors. a, HRTEM image of CdS/GeS core-shell heterostructure NW. **b**, EDS elemental mapping images of Cd, Ge, S. All the scale bars are 20 nm. **c**, XRD patterns of CdS NWs and as-constructed core-shell heterostructure NWs. **d**, Photodetection behaviors of pristine CdS NW and CdS/GeS core-shell heterostructure NW. **e**, Schematic for type-II CdS/GeS core-shell NWs photodetector.

In this work, a versatile strategy is exploited for the lattice-mismatch-free construction of III-V/chalcogenide core-shell heterostructure NWs by simply utilizing the surfactant and amorphous natures of chalcogenide semiconductors. Theoretically, this approach also can be used for lattice-mismatch-free construction of chalcogenide semiconductors core-shell heterostructure NWs. As shown in Fig. S18, the as-expected CdS/GeS core-shell heterostructure NW and the rational band alignment are successfully constructed. From the HRTEM image (Fig. S18a), the as-prepared NW shows apparent contrast between the core and shell. It is also obvious that the shell conformally wraps around the core. The clear lattice fringes are observed with the lattice spacings of 0.65 and 0.36 nm, indicating the good crystallinity of core NW. Noteworthy, no obvious crystal lattice fringes on the surface indicate the shell is amorphous. The elemental compositions of the core and shell are checked by EDS elemental mappings in Fig. S18b. The distribution of element Cd mainly concentrates in the core region, while elements Ge and S are observed in the whole NW, inferring that CdS/GeS core-shell heterostructure NW being successfully constructed. In the end, X-ray diffraction (XRD) further verifies the amorphous shell in Fig. S18c. Furthermore, the as-constructed CdS/GeS core-shell heterostructure NWs exhibit excellent photodetection performance with extended-spectrum detection range and much larger photocurrent compared to that of pristine

CdS NWs, as shown in Fig. S18d. The improved photodetection performance is attributed to the successful construction of type-II p-n core-shell heterostructure, as illustrated in Fig. S18e. Obviously, the developed lattice-mismatch-free construction strategy is efficient and versatile, not only for III-V NWs but also for chalcogenides.

Supplementary Table S1 Photodetection performances comparison of III-V core-shell heterostructure NWs.

**Core-shell NWs**	**Growth method**	**Heterostructure type**	**Photoresponse behavior**	***R* (A/W)**	***D** (Jones)**	**Response time (t_r/t_d)**	**Ref.**
GaAs/AlGaAs	MOCVD	Type-I	Positive	0.57 (@855 nm)	7.2×10^{10}		15
$GaAs_{1-x}Sb_x$ /InAs	MBE	Type- II p-n	Positive	0.12 (@1310 nm)	-	0.45 ms/0.86 ms (633nm)	16
InAs/AlSb	MBE	Type-II p-n	Negative	-	-	-	17
GaAs/AlGaAs	MOVPE	Type-I	Positive	10^{-4} (@800 nm)	-	5 ps	18
In-Rich InGaAs	MBE	-	Positive	5.75 (@1550 nm)	-	-	19
GaSb/GeS	CVD	Type-I p-p	Wavelength dependent bi-directional	400 (@1550 nm)	9.8×10^{10}	8.0 ms/12.0 ms (1550 nm)	This work
InGaAs/GeS	CVD	Type-II p-n	Positive	61.2 (@850 nm)	6.8×10^{11}	3.3 ms/2.9 ms (850 nm)	This work

Supplementary Table S2 Growth details of core-shell heterostructure NWs.

**Core-shell NWs**	**Catalyst (nm)**	**Growth of core NWs**					**Growth of shells**			
		**Source (g)**	**Sulfur (g)**	**S.T. (°C)**	**G.T. (°C)**	**Carrier gas (sccm)**	**Source (g)**	**S.T. (°C)**	**G.T. (°C)**	**Carrier gas (sccm)**
GaSb/GeS	Au/1.0	GaSb/0.4	0.4	750	560	H_2/200	GeS/0.1	500	320	H_2/200
GaAs/GeS	Au/1.0	GaAs/0.4	-	800	600	H_2/200	GeS/0.1	500	320	H_2/200
InGaAs /GeS	Ni/1.0	(InAs:GaAs =1:1) /0.4	-	810	610	H_2/200	GeS/0.1	500	320	H_2/200
GaSb/GeSe	Au/1.0	GaSb/0.4	0.4	750	560	H_2/200	GeSe/0.1	550	320	H_2/200
CdS/GeS	Au/1.0	CdS/0.1	-	750	540	Ar/50	GeS/0.1	500	320	H_2/200
GaSb/GaAs	Au/1.0	GaSb/0.4	0.4	750	560	H_2/200	GaAs/0.4	750	560	H_2/200

Note: S.T.: Source temperature; G.T.: Growth temperature; Growth substrate: Si/SiO_2; Pressure of CVD system: 6×10^{-3} Torr; Position of source and growth substrate: source powders of shell semiconductors (such as GeS, GeSe, and GaAs) are placed in upstream zone 1, source powders of core semiconductors (such as GaSb, GaAs, InGaAs, and CdS) are placed in midstream zone 2, the growth substrate is placed in the downstream zone 3, and sulfur powders are placed between zone 2 and 3.

Supplementary References

1. Gobeli, G. W. & Allen, F. G. Photoelectric properties of cleaved GaAs GaSb InAs and InSb surfaces-comparison with Si and Ge. *Phys. Rev.* **137**, A245-A254 (1965).
2. Jia, S. *et al.* Ultrahigh drive current and large selectivity in GeS selector. *Nat. Commun.* **11**, 4636 (2020).
3. Zhang, D. & Li, Z. InP/ZnS quantum dots functionalized AlGaAs/InGaAs open gate high electron mobility transistor. *J. Mater. Sci.: Mater. Electron.* **29**, 10663-10668, (2018).
4. Ahn, H.-W. *et al.* Effect of density of localized states on the ovonic threshold switching characteristics of

the amorphous GeSe films. *Appl. Phys. Lett.* **103**, 042908 (2013).

5. Hu, W. *et al.* Germanium/perovskite heterostructure for high-performance and broadband photodetector from visible to infrared telecommunication band. *Light Sci. Appl.* **8**, 106 (2019).
6. Wu, F. *et al.* High efficiency and fast van der Waals hetero-photodiodes with a unilateral depletion region. *Nat. Commun.* **10**, 4663 (2019).
7. Ma, Q. *et al*. Tuning ultrafast electron thermalization pathways in a van der Waals heterostructure. *Nat. Phys.* **12**, 455-460 (2016).
8. Fan, S. *et al.* Tailoring quantum tunneling in a Vanadium-doped $WSe_2/SnSe_2$ heterostructure. *Adv. Sci.* **7**, 1902751 (2020).
9. Lee, H. K. H.*et al.* The role of fullerenes in the environmental stability of polymer: fullerene solar cells. *Energy Environ. Sci.* **11**, 417-428 (2018).
10. Caprioglio, P. *et al.* On the origin of the ideality factor in perovskite solar cells. *Adv. Energy Mater.* **10**, 2000502 (2020).
11. Feng, S. *et al.* An ultrasensitive molybdenum-based double-heterojunction phototransistor. *Nat. Commun.* **12**, 4094 (2021).
12. Bonilla, R. S. & Wilshaw, P. R. A technique for field effect surface passivation for silicon solar cells. *Appl. Phys. Lett.* **104**, 232903 (2014).
13. Mallorqui, A. D. *et al*. Field-effect passivation on silicon nanowire solar cells. *Nano Res.* **8**, 673-681 (2015).
14. Lu, Z. *et al.* Ultrahigh speed and broadband few-layer $MoTe_2$/Si 2D-3D heterojunction-based photodiodes fabricated by pulsed laser deposition. *Adv. Funct. Mater.* **30**, 1907951 (2020).
15. Dai, X. *et al*. GaAs/AlGaAs nanowire photodetector. *Nano Lett.* **14**, 2688-2693 (2014).
16. Wang, X. *et al.* Vis-IR wide-spectrum photodetector at room temperature based on p-n junction-type $GaAs_{1-x}Sb_x$/InAs core-shell nanowire. *ACS Appl. Mater. Interfaces* **11**, 38973-38981 (2019).
17. Li, H. *et al.* Novel type-II InAs/AlSb core-shell nanowires and their enhanced negative photocurrent for efficient photodetection. *Adv. Funct. Mater.* **28**, 1705382 (2018).
18. Gallo, E. M. et al. Picosecond response times in GaAs/AlGaAs core/shell nanowire-based photodetectors. *Appl. Phys. Lett.* **98**, 241113 (2011).
19. Zhou, C. *et al.* Self-assembly growth of In-rich InGaAs core-shell structured nanowires with remarkable near-infrared photoresponsivity. *Nano Lett.* **17**, 7824-7830 (2017).
